# Identification of a novel germline frameshift mutation p.D300fs of PMS1 in a patient with hepatocellular carcinoma: A case report and literature review

**DOI:** 10.1097/MD.0000000000019076

**Published:** 2020-01-31

**Authors:** Xiaobin Li, Yuling Wu, Peisu Suo, Guifeng Liu, Lifeng Li, Xiaoni Zhang, Shifu Chen, Mingyan Xu, Lele Song

**Affiliations:** a HaploX Biotechnology, Co., Ltd.; b Department of Radiotherapy, the Eighth Medical Center of the Chinese PLA General Hospital, PR China.

**Keywords:** germline, HCC, hepatocellular carcinoma, MMR, PMS1, TACE

## Abstract

**Rationale:**

PMS1 is one of the mismatch repair (MMR) genes with potential crucial roles in carcinogenesis. Very few reports have been identified on germline PMS1 mutations with definite disease phenotype. Here we report a case of hepatocellular carcinoma (HCC) with a novel potential pathogenic germline PMS1 mutation.

**Patient concerns:**

A 46-year-old Chinese male with Hepatitis B infection history presented a single cancerous nodule (10×12×10 mm) at the left lobe of liver. The nodule was considered malignant by type-B ultrasonic and computed tomography (CT) examinations.

**Diagnosis and intervention:**

Liver lobectomy was performed to remove the liver cancerous nodule and postoperative TACE was performed for recurrence prevention. Pathological examination on resected tumor tissue confirmed the diagnosis of HCC. Whole-exome sequencing (WES) identified the c.900delT (p.D300fs) heterozygous germline mutation of PMS1, along with 253 nonsynonymous single nucleotide variations (SNVs), 14 Insertion or deletion mutations (INDELs) and 21 genes with copy number variations (CNVs). Three-dimensional prediction of protein tertiary structure suggested that the conformation of the enzyme active site and the ligand binding site might be changed due to the protein truncation.

**Outcomes:**

The patient was still alive in good condition with no sign of recurrence in 12 months follow-up period.

**Lessons:**

The affected pathways in this case were unique from previously reported HCC patients with no PMS1 germline mutations. The novel PMS1 germline mutation may increase cancer risk. The roles of PMS1 germline mutations in carcinogenesis need further investigation.

## Abstract 2

Supplemental Digital Content is available in the text

## 1 Introduction

Hepatocellular carcinoma (HCC) is one of the most common and fatal cancers worldwide, and it ranked the third most common cancer in China. It is well known that risk factors such as HBV/HCV chronic infection, alcoholic fatty liver disease and aflatoxin B1 exposure, contribute to the development of HCC.^[1]^ Many studies have reported the genomic investigation on somatic mutations, the abnormal pathways and potential mechanism in HCC that related to the above risk factors.^[2–6]^ However, the roles of germline mutations in HCC carcinogenesis have not been investigated in details.^[7]^

A few germline mutations related to HCC carcinogenesis have been reported recently.^[8–11]^ TERT promoter mutation might be involved in inherited HCC,^[9]^ and DICER1 mutation may be associated with familial recurrent liver tumors.^[10]^ Loss of germline PKM2 promoted HCC development.^[11]^ The most well-studied germline variations in cancer include the mutations of mismatch repair (MMR) genes, typically MLH1, MSH2, MSH6 and PMS2. They have been confirmed to connect with certain types of cancers, such as colorectal cancer and breast cancer.^[12–14]^ However, few studies have been reported on the correlation between germline MMR genes and HCC. Here a novel frameshift germline mutation (p.D300fs) of the PMS1 gene in a male HCC patient was identified via whole-exome sequencing. It exhibited unique mutation spectrum and pathways in promoting HCC compared with HCC without PMS1 germline mutations.

## 2 Ethics and methods

This study was approved by the hospital ethics committee, and written informed consent for sample collection, genetic testing and publication was obtained from the patient. Genomic DNA were extracted from FFPE tissue sample and peripheral blood sample using QIAamp DNA FFPE Tissue Kit (Qiagen, Shanghai, China) and TIANamp Blood DNA Kit (Tiangen Biotech, Beijing, China), respectively. WES was performed by the Haplox Biotechnology in Shenzhen on a NovaSeq 6000 sequencing platform (Illumina, San Diego, CA, USA). Sequencing data were mapped onto human hg19 reference genome, and germline and somatic variants including single-nucleotide polymorphisms, SNVs, INDELs and CNVs were identified using SAMtools, GATK, Mutect and annotated by ANNOVAR software, and gene fusions were identified by GeneFuse software.

## 3 Case report

### 3.1 Patient characteristics

A 46-year-old Chinese male with untreated hepatitis B history suffered from abdominal pain for months, and came to our hospital for examination. No abnormal results were found in blood routine test, blood biochemical test, liver function test and kidney function test. The levels of tumor biomarkers, including AFP, CEA and CA199 were within normal ranges. However, type-B ultrasonic and CT examinations revealed a nodule of 10×12×10 mm at the left lobe of his liver, and a malignant lesion was highly suspected. Hepatic lobectomy was performed to remove the primary tumor, and pathologic examination confirmed the diagnosis of HCC. The patient received postoperative TACE therapy following surgery, and no signs of recurrence or metastasis have been found 12 months after the therapy.

### 3.2 Genomic analysis

**Figure 1:**
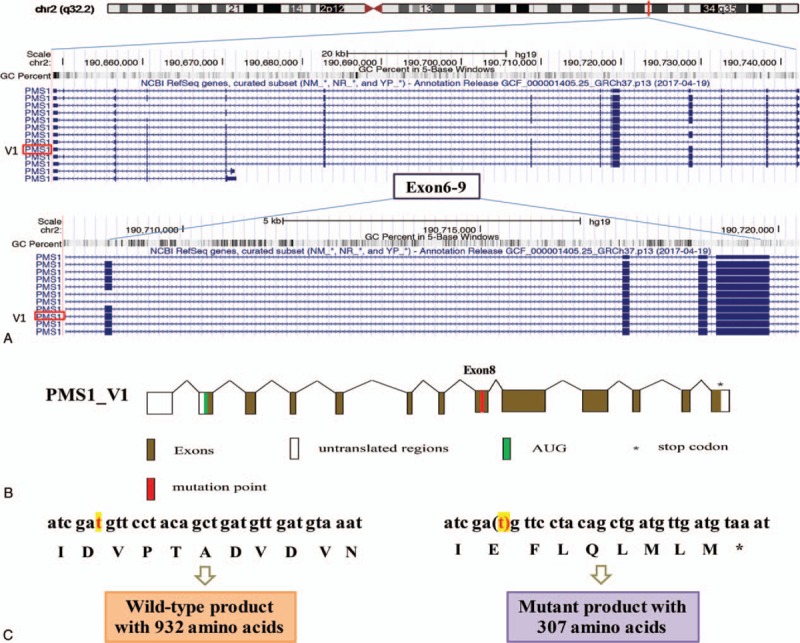
Genomic organization of the PMS1 gene. The UCSC genome browser (http://genome.ucsc.edu) was used to display the location and genomic organization of PMS1 on chromosome 2q32.2. (A) The position of PMS1 gene was highlighted and genomic region of Exon 6-9 covering approximately 10 kb is magnified. (B) Scheme of PMS1_V1 transcript including alternative splicing of exons. The AUG represents the start codon and the asterisk represents the stop codon. The mutation position is highlighted in red. Non-coding regions are indicated by open boxes and common coding open reading frames (ORF) are indicated by filled boxes. (C) Comparison of PMS1 wild type DNA sequence and the c.900delT (p.D300fs) sequence showed the generation of a stop codon and resulted in a mutant of 307 amino acids.

WES was carried out and germline mutations (supplementary table 1) and somatic mutations (supplementary table 2, 3 and 4 for lists of SNVs, INDELs and CNVs, respectively) were called and analyzed. Analysis of germline variations identified a novel, frameshift mutation of PMS1 (c.900delT, p.D300fs), which has not been reported in any database and publication (Fig. 1A and B). The deletion of T at position 900 led to the appearance of an inframe stop code (TAA) at position 923. This resulted in a truncated protein with only 307 amino acids, compared with the full wild type protein of 932 amino acids (Fig. 1C). Analysis of other MMR-related genes revealed several benign or likely benign germline variants, including MSH6 c.116G>A (benign), PMS2 c.1621A>G and c.1408C>T (both benign) MLH1 c.1151T>A (likely benign), MSH2 c.23C>T (like benign) and PMS2 c.2570G>C (likely benign), based on guidelines of the American College of Medical Genetics and Genomics (ACMG).

**Figure 2:**
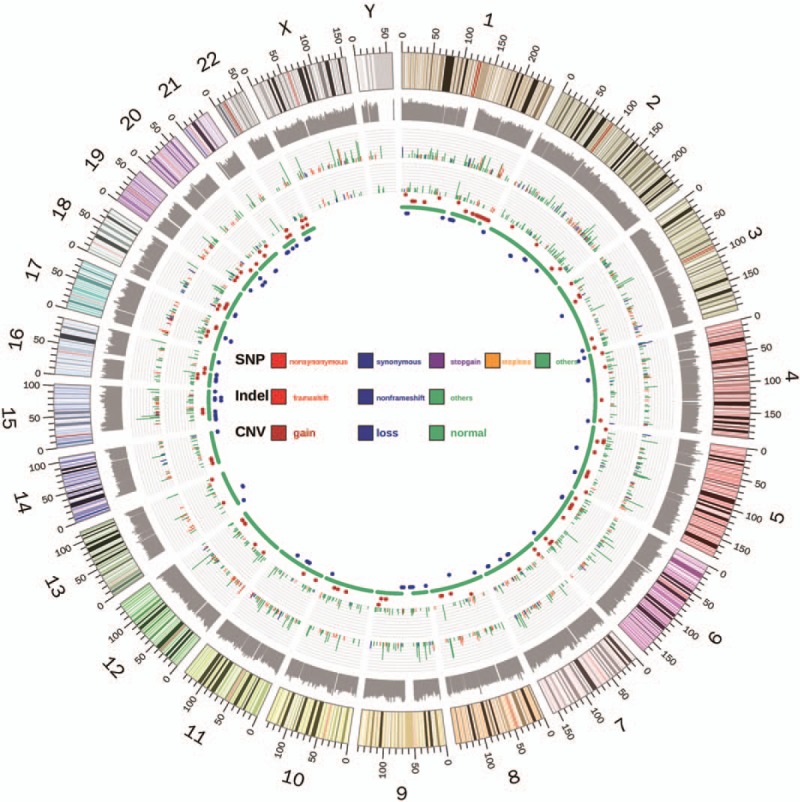
Circos scheme shows the whole-exome sequencing landscape of somatic SNV, Indel and CNV distribution of the tumor tissue in this study in 24 chromosomes. From outer to inner rings: the outermost ring shows the human genome scheme including 24 chromosomes, followed by log10 values of coverage depth in WES. The types and position of SNV and INDEL mutations are presented consecutively. The length of lines represents the variant allele frequency and the colors represent types of mutations. The innermost ring represents the CNV change, in which red dots stand for amplification and blue dots stand for deletion, while green stands for normal CNV.

**Figure 3:**
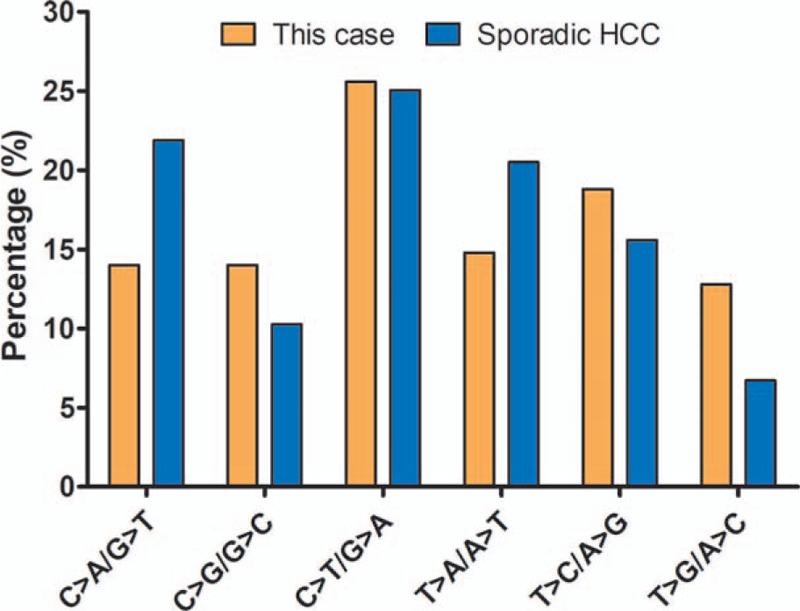
A comparison of the percentage of six types of base changes between this case (orange) and 10 sporadic HCC patients with no pathogenic or likely pathogenic germline mutations (blue).

**Figure 4:**
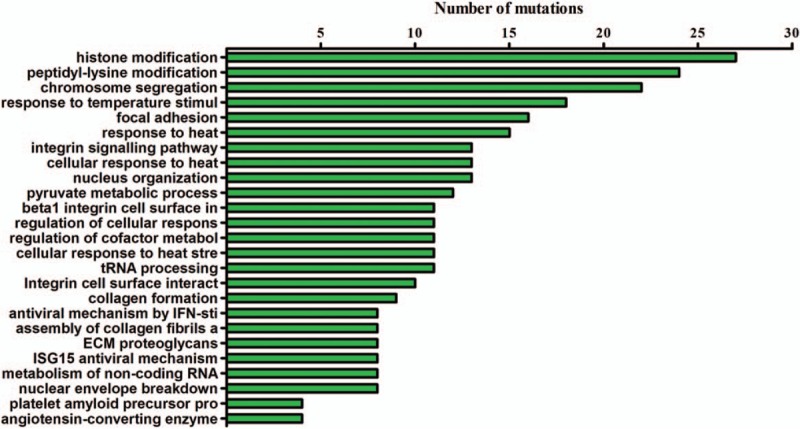
The results of the pathway enrichment analysis on the tumor tissue of this case. Analysis methods included GO term enrichment analysis and KEGG pathway enrichment analysis of mutated somatic genes. X axis indicates the numbers of mutations detected for each function or pathway.

The somatic mutations of the HCC tissue were called and analyzed. 253 SNV mutations (Fig. 2, Supplementary Table 2), 14 INDEL mutations (Fig. 2, Supplementary Table 3) and 24 CNVs (Fig. 2, supplementary table 4) were identified. The tumor mutation burden was 6.67 muts/Mb, with microsatellite stable (MSS) and PD-L1 expression level at 1%. One striking characteristic of the mutation spectrum was that the copy number amplification of several key driver genes (BRCA1, ERBB2, FGFR1, NTRK1, TERT and TP53) and several key functional genes (AKT2, ARID1A, CCND1, CDK4, FGF3, FGF4, FGFR3, IGF2, NOTCH1, PDGFRA, SRC, VEGFA, etc.) were observed. Mutation signatures were successfully obtained for this case, and compared with the mutation signature from a pool of 10 sporadic HCC patients without any known pathogenic or likely pathogenic germline mutations. The comparison in Figure 3 showed a strong predisposition for C>T/G>A mutation, and no statistically significant difference in the proportion of any type of base change was identified between this case and the 10 sporadic HCC patients (Fig. 3). Pathways enrichment analysis revealed that intracellular biological processes, mainly include histone modification, peptidyl-lysine modification and chromosome segregation, may be dysregulated in this HCC patient (Fig. 4).

### 3.3 Mutant structure and function prediction

**Figure 5:**
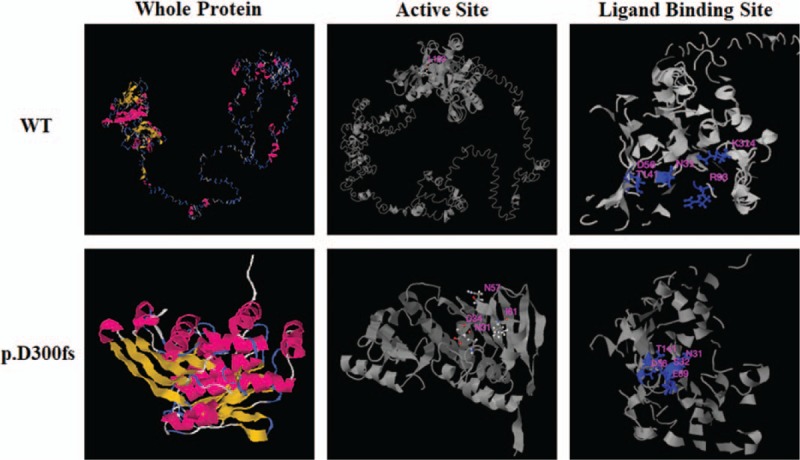
Prediction of the tertiary structure and function for wild type PMS1 and the p.D300fs mutant proteins with I-TASSER tool (https://zhanglab.ccmb.med.umich.edu/I-TASSER/). The tertiary structure (column ‘whole protein’), the prediction of enzyme active site (column ‘active site’) and the prediction of ligand binding site (column ‘ligand binding site’) of the both WT and mutant protein are shown as indicated.

In order to understand the potential alterations caused by PMS1 p.D300fs in protein structure and function, a tertiary protein structure and functional prediction were performed with the I-TASSER tool (https://zhanglab.ccmb.med.umich.edu/I-TASSER/) for wild type PMS1 and the p.D300fs mutant proteins. The whole protein was truncated with only 307 amino acids left from the N-terminus (Fig. 5 whole protein). The enzyme active site predicted for the wild type protein was at L123, while it was predicted to be at N31, D34, N57, I61 for the mutant (Fig. 5 active site). Furthermore, the ligand binding site for the wild type protein was predicted to be at N31, D65, R93, T141, K314, while it was predicted to be at N31, S32, D56, E59 and T141 for the mutant (Fig. 5 ligand binding site). It is clear from the prediction that the whole protein structure, the active site and the ligand binding site may be greatly altered by the p.D300fs mutant.

## 4 Discussion

HCC has emerged as a common malignancy worldwide and become a major cause of cancer-related death.^[15,16]^ Understanding the genetic basis of HCC has provided novel insights into carcinogenesis in the last decade.^[15,16]^ The most frequently mutated genes in HCC include TP53, RB1, CDKN2A, TERT, CTNNB1, VCX, AXIN1, FGF19, CNND1, RPS6KA3, etc.^[15–20]^ Meanwhile, studies have also revealed germline mutations associated with primary HCC, including HNF1α, BRCA2, TP53, MEN1 and TERT, etc.^[21,22]^ For example, Straub et al^[22]^ reported that germline mutation in hepatocyte nuclear factor 1α (HNF1α) accelerated malignant transformation into a well-differentiated HCC.

In this study, we identified a novel frameshift germline mutation of PMS1 (p.D300fs), however, this mutation has not been reported in any publications and databases. Since the mutation produced a truncated protein that was only approximately 1/3 length of the wild type PMS1 protein, we interpret the mutation as likely pathogenic based on ACMG guidelines. PMS1 is one of the MMR genes that play important roles in tumor occurrence, progression and prognosis. MMR system involves nine genes, including MLH1, MSH2, MSH3, MSH6, MLH3, PMS1, PMS2, MSH4 and MSH5,^[23]^ and the function and abnormalities of PMS1 and its mutations were not well studied. A study undertaken by Lipkin et al^[24]^ demonstrated that wild type PMS1 suppressed gastrointestinal tumorigenesis. Other reports^[25–28]^ have found PMS1 mutations were correlated with the susceptibility of hereditary non-polyposis colorectal cancer (HNPCC). TCGA database showed that the frequency of PMS1 variation was about 1.3% in HCC, including mutations of PMS1 E480Kfs∗36, V325I, M890 V and CNVs in two different gene coordinates (https://portal.gdc.cancer.gov/).

No PMS1 germline mutations have been reported in HCC, but a few have been reported in other cancers (Table 1). PMS1 (c.585_699del) was the first PMS1 germline mutation reported by Fraser et al in 1994,^[29]^ which was presumed to be responsible for the HNPCC phenotype. Betti et al recently reported a novel truncated germline PMS1 mutation (c.1380delT, p.Ser460fs) that may predispose malignant pleural mesothelioma.^[30]^ Other germline mutations of PMS1, mostly SNV mutations, were interpreted as benign or with unknown significance^[31–34]^ (Table 1). Interestingly, Yang et al^[35]^ revealed that PMS1 polymorphism sites of rs4920657, rs5743030, and rs5743100 were associated with the overall survival of rectal cancer patients who received postoperative chemoradiotherapy.^[35]^

**Table 1:**
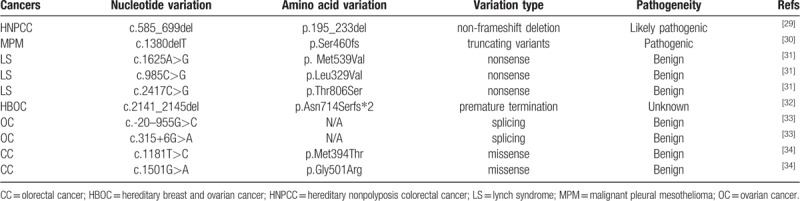
Summary of PMS1 germline mutations reported in literature.

The mutation spectrum and the corresponding aberrant pathways of this case were very different from those reported in sporadic HCC without PMS1 germline mutations. Expect one SNV mutation of TP53 (c.G701A), we did not find other nonsynonymous mutations in high frequently mutated genes in HCC, including TP53, RB1, CDKN2A, VCX, TERT, AXIN1, SELPLG, ALB, FGF19, CNND1, RPS6KA3, CTNNB1, CDKN2A, and CDKN1B.^[15]^ In contrast, copy number amplification of several key driver genes (BRCA1, ERBB2, FGFR1, NTRK1, TERT, and TP53) and several key functional genes (AKT2, ARID1A, CCND1, CDK4, FGF3, FGF4, FGFR3, IGF2, NOTCH1, PDGFRA, SRC, VEGFA, etc.) were observed. The genes amplified in this case were distinct from those reported in previous publications in HCC (MYC, RSPO2, CCND1, FGF19, VEGFA, CNND1, TERT),^[15,16]^ since driver gene amplifications were not commonly seen in these reports. A few CNVs of this case also have targeted drug implications, including the HER2 inhibitors for HER2 amplification and FGFR inhibitors for FGFR1 and FGFR3 amplification. Moreover, biological pathways are usually dysregulated in Wnt/β-catenin pathway, p53 pathway, RAS/RAF/MEK/ERK, PI3K/AKT/mTOR, Epithelial–mesenchymal transition (EMT), TGF-β and Notch signaling in HCC without PMS1 germline mutations.^[16,18]^ However, biological functions altered in this case were not common in HCC. These observations suggest that the genomic aberrances in this case were quite unique, and it can be speculated that the specific mutant of PMS1 (p.D300fs) may lead to uncommon abnormalities of the case.

The mutational spectrum is usually dominated by C>T substitution in cancers carrying MMR gene mutations.^[17,36]^ Fang et al proposed that the loss-of-function mutations within PMS1 increased mutation frequency and promoted tumorigenesis.^[37]^ Our observation in this study suggested that sporadic HCC without germline pathogenic or likely pathogenic mutations also exhibited a predisposition of C>T substitution. Consistent with previous reports, our case also indicated a strong predilection for C > T transition. This suggested that although the mutated gene and pathways of this case were quite different from those of previously reported HCC, the base alteration was very similar. We would speculate that this patient may share similar mechanism of carcinogenesis to other MMR-deficient carcinomas.

Although we thoroughly examined the genetic alterations in this patient, it remains to be answered whether PMS1 (p.D300fs) alone is sufficient to induce cancer. The mechanism of PMS1 (p.D300fs) causing distinct somatic mutation alterations and biological pathway dysregulations is also worth more investigation. These may need *in vitro* experiments on protein and cellular functions and transgenic studies using animal models.

## 5 Conclusions

In conclusion, we reported the first HCC case harboring a novel PMS1 germline mutation, and identified distinct somatic mutation spectrum and biological pathways from previous reports. Our study may contribute to the understanding of molecular mechanisms of hepatic carcinogenesis and the therapeutic options in the background of potential pathogenic germline mutations.

## Acknowledgments

We thank the Shenzhen Science and Technology Committee and the Shenzhen Leading Group for the Development of Emerging High-tech Industries for funding support.

## Author contributions

**Conceptualization:** Xiaobin Li, LELE SONG.

**Data curation:** Yuling Wu, Lifeng Li, LELE SONG.

**Formal analysis:** Xiaobin Li, Yuling Wu, Peisu Suo, Lifeng Li, LELE SONG.

**Funding acquisition:** LELE SONG.

**Investigation:** Yuling Wu, Guifeng Liu, LELE SONG.

**Methodology:** Peisu Suo.

**Project administration:** Xiaobin Li, Guifeng Liu, LELE SONG.

**Resources:** Guifeng Liu.

**Software:** Yuling Wu, Lifeng Li.

**Supervision:** Xiaobin Li, Lifeng Li, LELE SONG.

**Validation:** Peisu Suo.

**Writing – original draft:** Xiaobin Li, LELE SONG.

**Writing – review & editing:** Xiaobin Li, LELE SONG.

LELE SONG orcid: 0000-0003-0296-2978.

## Supplementary Material

Supplemental Digital Content

## Supplementary Material

Supplemental Digital Content

## Supplementary Material

Supplemental Digital Content

## Supplementary Material

Supplemental Digital Content
